# Suicide—Life Assurance

**Published:** 1848-04-01

**Authors:** 

**Affiliations:** London


### Correspondence.

### SUICIDE—LIFE ASSURANCE.

(*To the Editor of the Journal of Psychological Medicine.*)

SIR,—Allow me to direct the attention of your readers to a recent decision in the Exchequer Chamber, in the cause of *Clift* v. *Schwabe.* The report of this case will be found in the "Law Journal," published on the 1st of last January. I need only state, for the benefit of your lay readers, that the question raised for the opinion of the judges, was substantially, *whether a policy of life insurance, which excludes the risk of the assured's "committing suicide," ought to be construed as intending to exclude all self-killing, whether the party may at the time have been compos mentis, or the reverse.* It would be foreign to the character of your work to enter into the argument on the legal construction of such a contract; but the decision of the majority of the judges is so directly opposed to the right principles, that I feel compelled to express my repectful dissent. It is with no slight satisfaction that I find myself borne out in this dissent by such high authorities as Chief Baron Pollock, Mr. Justice Wightman, and Mr. Justice Cresswell. As I am not writing for the exclusive reading of lawyers, I may be allowed to express in popular language, that this judicial decision virtually excepts the suicide of an insane man from the risks of disease, against which insurance offices profess to indemnify, unless such fatal termination of the malady is specially provided for by the contract of insurance. This would be stating the position too broadly, if I were writing with legal accuracy; but it is sufficiently accurate for the present purpose.

If anything were wanting to show the great importance of cultivating a more correct and scientific knowledge of psychology, it would be found in the singular circumstance, that throughout the argument of counsel or the several judgments of the bench, there is not, except in the elaborate opinion of the Chief Baron, the least allusion to the fact, that insanity is, in truth, disease; and as disease, necessarily within the contemplation of parties to an insurance. Suicide, where intentional, and not accidental, (as by the inadvertent taking of poison, or the unskilful use of a gun,) must necessarily be the act of a sound or an unsound mind; if the former, it is felonious; if the latter, it results from disease, and like gout, epilepsy, or intemperate habit, must be a risk contemplated by a life policy, unless specially excepted. It seems unaccountable that a majority of the bench should have arrived at a different conclusion, (for their decision on the meaning of the word "suicide" amounts to this,) if they had rightly understood the intimate and invariable connexion between mania and bodily disease. Their inexperience on this subject has led to a decision which exempts insurance offices who have inserted in their policies a protecting clause against "suicide" in general terms, from one of the most serious of all the risks against which they propose to indemnify their customers.

It is well known, at least to medical men, that mania tends to shorten life, independently of suicidal hazard; but surely, it would never be contended, that in the absence of any special exception, a policy effected on the life of a sane man would become void if he subsequently fell a victim to a brain fever! or even if, in a paroxysm of frenzy, he threw himself on the fire, and received fatal injury. Yet wherein does this differ from the risk of his discharging a pistol at his head, under the irrational impulse that it would relieve the agony of his brain?

Chief Baron Pollock used these arguments with his learned brethren in vain; but I suspect that they will, nevertheless, induce a strong feeling in the public mind, when the operation of this decision becomes practically felt, that our bench, no less than our juries, require to be a little enlightened on the nature of insanity. "In my judgment," says the Chief Baron, "if death be the result of disease, whether by affecting the senses or the reason, the insurance office is liable under this policy." I entirely agree with his lordship. But what can be said to an interlocutory remark of one of the most learned on the bench—"Surely there are degrees of insanity?" There are varieties of insanity; there are stages of insanity; there are many forms of insanity, and its phases are as different as its causes; but insanity, where unequivocal and rael, admits of no graduation; the mind is sound or unsound, subject to our former doubt whether, abstractedly and metaphysically speaking, the mind, as such, is susceptible of disease. If this doubt is not philosophically well founded, then it is as absurd to speak of graduated insanity, as of graduated paralysis or graduated gout. All morbid affection is progressive, and in its different stages may be more or less painful, more or less dangerous; but disease, in any scientific sense, is an antagonistic term to health, and admits of no measure by a graduated scale. The science of pathology is sufficiently obscure already. One diseased action, in itself comparatively unimportant, will often elicit other morbid action hitherto dormant in the constitution, of serious and even fatal tendency. Thus, as every medical tyro is aware, syphilis will bring out a latent scrofulous affection. But if it is required to define with legal accuracy the exact stages at which disease may assume the character of ultimate danger, it implies a nicety of discrimination which we do not hesitate to say is not attainable in the present state of pathological science. This is not less true of insanity than of any other malady or organic derangement well developed. Medical experience can assign no definite limits within which it can be declared exempt from consequential risk.

I am, your obedient servant,

A Jurist.

*London, March.*

---

